# Seasonal Variation for Plantar Fasciitis: Evidence from Google Trends Search Query Data

**DOI:** 10.3390/healthcare10091676

**Published:** 2022-09-02

**Authors:** Seok-Min Hwang, Seok Kim, Suk-Hyun Hwang

**Affiliations:** Department of Orthopedic Surgery, Seoul Red Cross Hospital, 9, Saemunan-ro, Jongno-gu, Seoul 03181, Korea

**Keywords:** plantar fasciitis, heel pain, internet search, seasonality

## Abstract

We aimed to determine the seasonal trends in internet searches for plantar fasciitis and related symptoms in various countries using search engine query data on Google. We used Google Trends to obtain internet search query data from January 2009 to December 2019. We collected monthly search volumes for the query terms “plantar fasciitis” and “heel pain” in the USA, Canada, the U.K., Ireland, Australia, and New Zealand. Statistical analysis of the seasonal effects on plantar fasciitis was performed using a cosinor model. The cosinor analyses confirmed statistically significant seasonal patterns in the relative search volumes for the terms “plantar fasciitis” and “heel pain” in the USA, Canada, the U.K., Ireland, and Australia, with peaks during the summer and troughs during the winter. For New Zealand, the seasonal trend was statistically significant only for the term “plantar fasciitis”, while a similar trend for the term “heel pain” was present without achieving statistical significance for seasonality. This seasonality is thought to be related to more frequent occurrence of plantar fasciitis due to increased physical activity of people during the warmer months. In this study, the search query data using the terms “plantar fasciitis” and “heel pain” on Google Trends show significant seasonal variation across several countries, with a peak in the summer and a trough in the winter.

## 1. Introduction

Plantar fasciitis is the most common cause of heel pain. Over the course of their lifetime, about 10% of people experience a common disease [1,2]. Plantar fasciitis accounts for about 11–15% of all foot symptoms that need medical treatment in adults. The number of patients having plantar fasciitis accounted for about 1% of all orthopedic visits [3]. Additionally, a survey of 2955 members of the American Podiatric Medical Association revealed that heel pain/plantar fasciitis was the most common foot condition being treated in podiatric clinics [4]. The mechanisms underlying plantar fasciitis are complex and multifactorial [5]. Patient-related factors include high body mass index (BMI), increased foot pronation, advanced age, and limited ankle dorsiflexion. Physical factors include running activities, standing for prolonged periods, and increased activity level [1,2,6,7,8]. These factors lead to pathologic overload at the calcaneal insertion of the plantar fascia, causing degenerative changes that include microtrauma in the fascia, necrosis of collagen, myxoid degeneration, and angiofibroblastic hyperplasia [1,9].

Various epidemiological studies have associated plantar fasciitis with age, sex, BMI, weight-bearing activities (such as walking or standing), and foot biomechanics [6,7,8]. However, there are few studies associating plantar fasciitis with seasons. A retrospective cohort study, using the Dutch primary care database from January 2013 to December 2016, showed an increased incidence of plantar fasciitis during September and October and a decrease in the winter months [10]. Another study, analyzing the Korean Health Insurance Review & Assessment Service database from January 2016 to December 2019, showed the highest incidence of plantar fasciitis cases in August and the lowest in February among the South Korean population [11]. Although the above studies reported seasonal effects on plantar fasciitis, they were limited to specific populations.

Nowadays, most people can easily and quickly find health information by entering an appropriate search term in an internet search engine [12]. Therefore, the internet has become an important resource for patients and clinicians to obtain health and medical-related information [13]. Big data generated through internet portal sites are being used in health and medical research. It is expected that internet search-based data can complement traditional data acquisition methods, such as patient research and medical record verification. Internet search query data have helped in the confirmation of disease epidemiology, health-related behaviors, and public interest [13,14,15]. When people experience physical abnormalities, pain, or are diagnosed with illness, they frequently obtain medical information through internet searches. In order to use internet search volume data in health-related research, it is important to evaluate how well the internet data reflect the actual severity or prevalence of diseases in real time. Several studies have reported significant correlations between the internet search volume data for various diseases and their actual occurrence status [16,17,18,19,20,21]. Furthermore, studies have confirmed the seasonality of internet searches for various diseases through internet search volume data [22,23,24,25,26]. The traditional survey method for checking the seasonality of disease is time-consuming, labor-intensive, and expensive. In comparison, the novel survey method of using internet search volume data is faster, easier, cheaper, and obtained in real time [12,27]. Thus, this new approach could effectively complement traditional data acquisition methods.

To our knowledge, no study has determined the seasonality of plantar fasciitis using the internet search query data. Therefore, the purpose of the present study was to evaluate the seasonal variation in plantar fasciitis and related symptoms in various countries using the search volume data from Google Trends.

## 2. Materials and Methods

### 2.1. Internet Tool and Search Criteria

We used Google Trends data to examine the volume of plantar fasciitis search queries on the internet. Google Trends is a publicly and freely accessible data service provided by Google Inc. (Mountain View, CA, USA) that shows the relative search volume (RSV) of a specific term used in their search engine. Google Trends computes the relative proportion of searches for any given keyword out of all searches performed on Google over a specific time period in a particular region [28]. The RSV has a range from 0 to 100 and anyone is free to download it. An RSV value of 100 represents the maximum volume of a specific search term, and 0 indicates that the search query is at less than 1% of its peak popularity [26]. Google Trends automatically eliminates repeated searches by the same user within a short period of time to avoid selection bias. It provides results filtered by time, region, category, and search type [28].

Heel pain is the most common symptom of plantar fasciitis. In order to examine the seasonality of plantar fasciitis, we searched for the query terms “plantar fasciitis” and “heel pain” on Google Trends and exported the monthly search volume data. These queries were searched within the USA, Canada, the U.K., Ireland, Australia, and New Zealand from January 2009 to December 2019, as these countries represent the native English-speaking populations in the northern and southern hemispheres.

### 2.2. Statistical Analysis

We performed a cosinor analysis to assess the seasonality of plantar fasciitis-related search volumes, similar to prior studies that examined the seasonal variation in other health problems using Google Trends [22,23,24,25]. Barnett et al. detailed the statistical method of the cosinor analysis and software usage in their study [29]. In short, the cosinor analysis determines potential seasonal patterns using a sinusoidal equation that estimates the amplitude (A, size of the seasonal changes), phase month (P, occurrence of the seasonal peak), and length of the seasonal cycle (established at 12 for monthly data). Because the sinusoid is part of a generalized linear model, the statistical significance of any seasonal trend can be calculated. The cosinor analysis has two expressions, a sine and a cosine expression, and presents two *p*-values, a sine and a cosine *p*-value. Both *p*-values were assessed for statistical significance. The significance level was established at *p* < 0.025 to reduce the false discovery rate by multiple tests. All statistical analyses were conducted using the R version 4.1.0 (R Foundation for Statistical Computing, Vienna, Austria) software package.

## 3. Results

**Figure 1:**
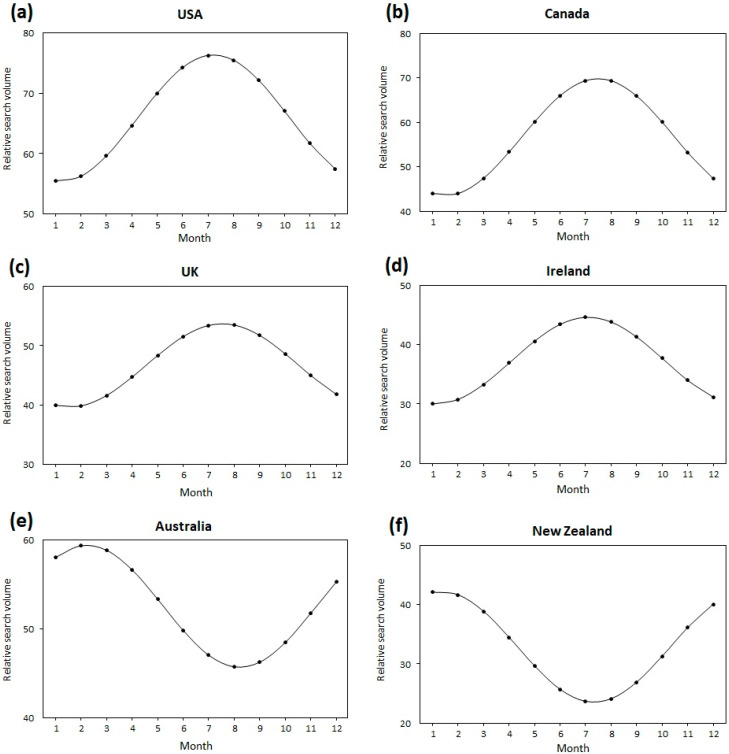
The plots of the cosinor model for seasonality in Google Trends search volume data for “plantar fasciitis” in (**a**) the USA, (**b**) Canada, (**c**) the U.K., (**d**) Ireland, (**e**) Australia, and (**f**) New Zealand. The numbers correspond to the months as follows: 1 = January; 2 = February; 3 = March; 4 = April; 5 = May; 6 = June; 7 = July; 8 = August; 9 = September; 10 = October; 11 = November; 12 = December.

**Figure 2:**
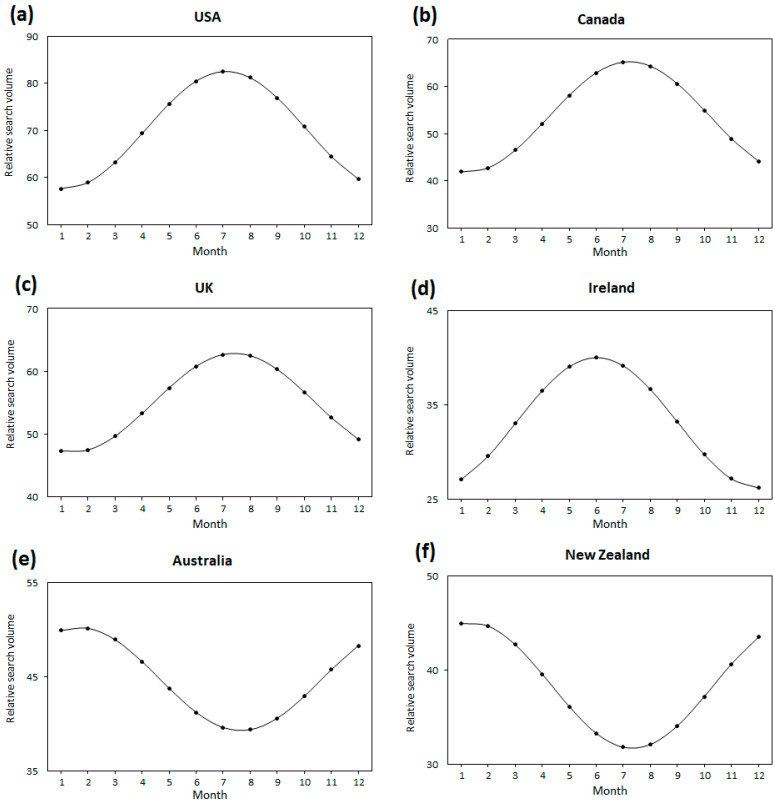
The plots of the cosinor model for seasonality in Google Trends search volume data for “heel pain” in (**a**) the USA, (**b**) Canada, (**c**) the U.K., (**d**) Ireland, (**e**) Australia, and (**f**) New Zealand. The numbers correspond to the months as follows: 1 = January; 2 = February; 3 = March; 4 = April; 5 = May; 6 = June; 7 = July; 8 = August; 9 = September; 10 = October; 11 = November; 12 = December.

The results of the cosinor analysis are presented in Table 1 and Table 2, and the plots are shown in Figure 1 and Figure 2. The cosinor analyses confirmed statistically significant seasonal patterns in the RSV for the term “plantar fasciitis” in the USA [A = 10.45, P = 7.22, low point month (L) = 1.22; *p* < 0.001], Canada (A = 13.14, P = 7.49, L = 1.49; *p* < 0.001), the U.K. (A = 7.03, P = 7.54, L = 1.54; *p* < 0.001), Ireland (A = 7.32, P = 7.11, L = 1.11; *p* = 0.007), Australia (A = 6.86, P = 2.22, L = 8.22; *p* = 0.004), and New Zealand (A = 9.34, P = 1.33, L = 7.33; *p* < 0.001; Figure 1). Search peaks were observed during summer (month of July in countries of the northern hemisphere, February in Australia, and January in New Zealand) and troughs during the winter months (January in countries of the northern hemisphere, August in Australia, and July in New Zealand). Comparing countries of the northern to the southern hemisphere, notably, the peaks in summer and troughs in winter were out of phase by 6 months.

The cosinor analysis showed statistically significant seasonal effect patterns in the RSV of “heel pain” in the USA (A = 12.45, P = 7.11, L = 1.11; *p* < 0.001), Canada (A = 11.75, P = 7.23, L = 1.23; *p* < 0.001), the U.K. (A = 7.86, P = 7.42, L = 1.42; *p* < 0.001), Ireland (A = 6.91, P = 6.02, L = 12.02; *p* = 0.01), and Australia (A = 5.47, P = 1.66, L = 7.66; *p* = 0.002). Although it showed a similar trend for New Zealand, statistical significance was not met (A = 6.69, P = 1.34, L= 7.34; *p* = 0.07; Figure 2). In summary, search peaks were found in the summer months (July in the USA, Canada, and the U.K.; June in Ireland; January in Australia and New Zealand) and troughs in the winter months (January in USA, Canada, and the U.K.; December in Ireland; July in Australia and New Zealand). This trend was similar to that observed for the term “plantar fasciitis”.

**Figure 3:**
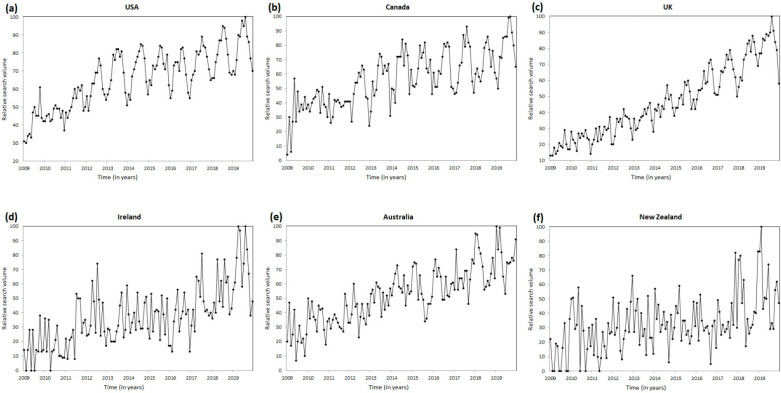
Time series plots for Google Trends search volume data for “plantar fasciitis” from January 2009 to December 2019 in (**a**) the USA, (**b**) Canada, (**c**) the U.K., (**d**) Ireland, (**e**) Australia, and (**f**) New Zealand.

**Figure 4:**
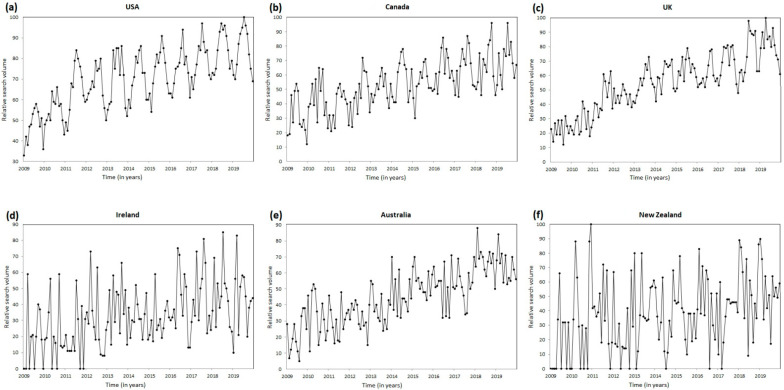
Time series plots for Google Trends search volume data for “heel pain” from January 2009 to December 2019 in (**a**) the USA, (**b**) Canada, (**c**) the U.K., (**d**) Ireland, (**e**) Australia, and (**f**) New Zealand.

The consistency of seasonal variation over the entire period, identified by the cosinor analysis, is shown as time series plots (Figure 3 and Figure 4). In summary, the RSV for the terms “plantar fasciitis” (Figure 3) and “heel pain” (Figure 4) on the Google search engine steadily increased from January 2009 to December 2019 in the USA, Canada, the U.K., Ireland, Australia, and New Zealand.

## 4. Discussion

To our best knowledge, this study is the first to analyze the seasonality of plantar fasciitis across several countries using internet search query data. Our results showed a significant seasonal variation in Google searches for “plantar fasciitis” and “heel pain” in the USA, Canada, the U.K., Ireland, Australia, and New Zealand, with peaks in summer and troughs in winter. The data are representative of both hemispheres because of the countries selected for this study. The seasonal variation for the frequency of the search terms in the northern hemisphere countries is comparable to the southern ones with opposite meteorological months. Hence, the peaks during summer and troughs during winter were out of phase by about 6 months between the hemispheres, suggesting that the variations were seasonal, rather than calendar-driven.

While the mechanisms underlying the seasonal trends of plantar fasciitis detected in this study cannot be investigated, previous studies have assessed the seasonality of plantar fasciitis [10,11]. Although the present study was conducted using internet search data and is thus inadequate for the direct comparison of seasonal trends with the incidence of plantar fasciitis, our findings are consistent with those of previous studies.

Several factors contribute to the development of plantar fasciitis. Among them, a factor having seasonality is an increase in physical activity. Therefore, we hypothesize that the seasonal trend of plantar fasciitis may be associated with increased activity such as long walks or running during the warmer months. Tucker and Gilliland, based on a systematic review of 37 studies comprising 291,883 people from eight countries, concluded that physical activity was indeed affected by seasons and weather [30]. The level of physical activity was the highest during spring and summer (April–August) and tended to decrease in the winter. Most studies have confirmed that physical activity peaks in July and August. Accordingly, it is plausible that the internet-based search for “plantar fasciitis” trending highest in summer and lowest in winter directly corresponds to the increased physical activity in summer and its decrease in winter, respectively.

In recent years, there has been increased use of the internet to find information related to symptoms and diseases. Hence, clinical and health policy researchers are increasingly interested in internet search data, particularly Google Trends [13,14,15]. According to PubMed search, original research articles or letters related to Google Trends have increased by more than 20 times from 2009 to 2018 [14]. Previous studies have reported the correlation between internet search query data on various health problems and the data on the actual incidence of a disease [16,17,18,19,20,21]. These investigations confirmed that the online search volume data can significantly reflect the actual occurrence status of health problems. Based on the clinical usefulness of the internet search volume, several studies using Google Trends have shown seasonal trends of internet searches for various diseases, including restless legs syndrome, snoring and sleep apnea, gout, psoriasis, and cellulitis [22,23,24,25,26]. We investigated whether seasonal variations exist in the internet search volume related to plantar fasciitis in several countries. Using Google Trends-based internet search volume during the period from January 2009 to December 2019, we found a seasonality of plantar fasciitis across different geographic areas, with a peak in the summer months.

The present study, however, has some limitations that must be considered when interpreting the results. First, Google Trends data do not provide the demographics of the people who perform the keyword-based internet search. Hence, important inter-individual differences, such as age, education level, and income, associated with the probability of utilizing web-based health information cannot be evaluated. Not all users who search the internet reflect the actual patient population; hence, it is incorrect to generalize the results. Second, the number of countries analyzed was limited. A more representative result could be obtained if more countries were analyzed from different cultures and locations, and if we are able to investigate how differences in climate, race, and social conditions affect the occurrence of plantar fasciitis. We consider it necessary to assess socio-cultural differences through additional analysis of different countries. Third, the act of searching for health-related information on the internet can occur even without the symptom in an affected individual, such as during media coverage of the illness, social health campaigns, outbreaks of the illness among celebrities, and for academic or research purposes. However, this study did not identify specific situations that could affect such searches. Fourth, the internet search engine used in this study was only Google. Therefore, there may be a selection bias toward people who chose to use Google. Nevertheless, this limitation is somewhat compensated by the fact that Google holds more than 80% of all search engine market shares in the countries considered for this study [31]. Finally, the study did not explain possible mechanisms underlying the observed seasonal variation in internet searches for plantar fasciitis. Therefore, additional clinical studies are needed to determine whether season has any clinical association with plantar fasciitis.

## 5. Conclusions

In conclusion, the search data of query terms for “plantar fasciitis” and “heel pain” on Google Trends show significant seasonal variation across several countries, with a peak in the summer and a trough in the winter. The present study provides another line of evidence for the seasonal trend of plantar fasciitis. It shows that Google searches for plantar fasciitis have steadily increased in recent years. Further research is required to reveal the potential mechanism underlying the seasonality of plantar fasciitis and the implication of these seasonal effects on clinical practice.

## Figures and Tables

**Table 1:** Country-wise seasonal variations in search volume data for “plantar fasciitis” using Google Trends.

Country	Amplitude (A)	Phase Month (P)	Low Point Month (L)	*p*-Value
USA	10.45	7.22	1.22	<0.001
Canada	13.14	7.49	1.49	<0.001
U.K.	7.03	7.54	1.54	<0.001
Ireland	7.32	7.11	1.11	0.007
Australia	6.86	2.22	8.22	0.004
New Zealand	9.34	1.33	7.33	<0.001

**Table 2:** Country-wise seasonal variations in search volume data for “heel pain” using Google Trends.

Country	Amplitude (A)	Phase Month (P)	Low Point Month (L)	*p*-Value
USA	12.45	7.11	1.11	<0.001
Canada	11.75	7.23	1.23	<0.001
U.K.	7.86	7.42	1.42	<0.001
Ireland	6.91	6.02	12.02	0.01
Australia	5.47	1.66	7.66	0.002
New Zealand	6.69	1.34	7.34	0.07

## Data Availability

The data presented in this study are available upon request from the corresponding author.

## References

[1] Neufeld S.K., Cerrato R. (2008). Plantar fasciitis: Evaluation and treatment. J. Am. Acad. Orthop..

[2] Crawford F., Thomson C. (2003). Interventions for treating plantar heel pain. Cochrane Database Syst. Rev..

[3] Riddle D.L., Schappert S.M. (2004). Volume of ambulatory care visits and patterns of care for patients diagnosed with plantar fasciitis: A national study of medical doctors. Foot Ankle Int..

[4] Al Fischer Associates, Inc (2003). 2002 Podiatric Practice Survey. Statistical results. J. Am. Podiatr. Med. Assoc..

[5] Schillizzi G., Alviti F., D’Ercole C., Elia D., Agostini F., Mangone M., Paoloni M., Bernetti A., Pacini P., Polti G. (2021). Evaluation of plantar fasciopathy shear wave elastography: A comparison between patients and healthy subjects. J. Ultrasound.

[6] Riddle D.L., Pulisic M., Pidcoe P., Johnson R.E. (2003). Risk factors for Plantar fasciitis: A matched case-control study. J. Bone Joint Surg. Am..

[7] Sobhani S., Dekker R., Postema K., Dijkstra P.U. (2013). Epidemiology of ankle and foot overuse injuries in sports: A systematic review. Scand. J. Med. Sci. Sports.

[8] Rhim H.C., Kwon J., Park J., Borg-Stein J., Tenforde A.S. (2021). A Systematic Review of Systematic Reviews on the Epidemiology, Evaluation, and Treatment of Plantar Fasciitis. Life.

[9] Lemont H., Ammirati K., Usen N. (2003). Plantar fasciitis. A degenerative process (fasciosis) without inflammation. J. Am. Podiatr. Med. Assoc..

[10] Rasenberg N., Bierma-Zeinstra S.M., Bindels P.J., Lei J.V.D., Middelkoop M.V. (2019). Incidence, prevalence, and management of plantar heel pain: A retrospective cohort study in Dutch primary care. Br. J. Gen. Pract..

[11] Hwang S.M., Lee G.H., Oh S.Y. (2021). Correlation between Internet Search Query Data and the Health Insurance Review & Assessment Service Data for Seasonality of Plantar Fasciitis. J. Korean Foot Ankle Soc..

[12] Cervellin G., Comelli I., Lippi G. (2017). Is Google Trends a reliable tool for digital epidemiology? Insights from different clinical settings. J. Epidemiol. Glob. Health.

[13] Nuti S.V., Wayda B., Ranasinghe I., Wang S., Dreyer R.P., Chen S.I., Murugiah K. (2014). The use of google trends in health care research: A systematic review. PLoS ONE.

[14] Arora V.S., McKee M., Stuckler D. (2019). Google Trends: Opportunities and limitations in health and health policy research. Health Policy.

[15] Carr L.J., Dunsiger S.I. (2012). Search query data to monitor interest in behavior change: Application for public health. PLoS ONE.

[16] Xu C., Yang H., Sun L., Cao X., Hou Y., Cai Q., Jia P., Wang Y. (2020). Detecting Lung Cancer Trends by Leveraging Real-World and Internet-Based Data: Infodemiology Study. J. Med. Internet Res..

[17] Chen Y., Zhang Y., Xu Z., Wang X., Lu J., Hu W. (2019). Avian Influenza A (H7N9) and related Internet search query data in China. Sci. Rep..

[18] Bahk G.J., Kim Y.S., Park M.S. (2015). Use of Internet Search Queries to Enhance Surveillance of Foodborne Illness. Emerg. Infect. Dis..

[19] Cho S., Sohn C.H., Jo M.W., Shin S.Y., Lee J.H., Ryoo S.M., Kim W.Y., Seo D.W. (2013). Correlation between national influenza surveillance data and google trends in South Korea. PLoS ONE.

[20] Woo H., Cho Y., Shim E., Lee C.G., Kim S.H. (2016). Estimating Influenza Outbreaks Using Both Search Engine Query Data and Social Media Data in South Korea. J. Med. Internet Res..

[21] Shin S.Y., Seo D.W., An J., Kwak J., Jo M.W. (2016). High correlation of Middle East respiratory syndrome spread with Google search and Twitter trends in Korea. Sci. Rep..

[22] Ingram D.G., Plante D.T. (2013). Seasonal trends in restless legs symptomatology: Evidence from Internet search query data. Sleep Med..

[23] Ingram D.G., Matthews C.K. (2015). Plante, D.T.; Seasonal trends in sleep-disordered breathing: Evidence from internet search engine query data. Sleep Breath..

[24] Kardeş S. (2019). Seasonal variation in the internet searches for gout: An ecological study. Clin. Rheumatol..

[25] Kardeş S. (2019). Seasonal variation in the internet searches for psoriasis. Arch. Dermatol. Res..

[26] Zhang X., Dang S., Ji F., Shi J., Li Y., Li M., Jia X., Wan Y., Bao X., Wang W. (2018). Seasonality of cellulitis: Evidence from Google Trends. Infect. Drug Resist..

[27] Zhang W., Yan K., Shen D. (2021). Can the Baidu Index predict realized volatility in the Chinese stock market?. Financ. Innov..

[28] Google Trends Help Center. https://support.google.com/trends/.

[29] Barnett A.G., Baker P., Dobson A.J. (2012). Analysing Seasonal Data. R J..

[30] Tucker P., Gilliland J. (2007). The effect of season and weather on physical activity: A systematic review. Public Health.

[31] Statcounter Global Stats—Search Engine Market Share Worldwide. https://gs.statcounter.com/search-engine-market-share/.

